# Biodiversity of *Klebsormidium* (Streptophyta) from alpine biological soil crusts (Alps, Tyrol, Austria, and Italy)

**DOI:** 10.1111/jpy.12316

**Published:** 2015-07-14

**Authors:** Tatiana Mikhailyuk, Karin Glaser, Andreas Holzinger, Ulf Karsten

**Affiliations:** ^1^ M.H. Kholodny Institute of BotanyNational Academy of Science of UkraineTereschenkivska St. 2KievUA‐01001Ukraine; ^2^ Institute of Biological Sciences, Applied Ecology and PhycologyUniversity of RostockAlbert‐Einstein‐Strasse 3RostockD‐18057Germany; ^3^ Institute of Botany, Functional Plant BiologyUniversity of InnsbruckSternwartestrasse 15InnsbruckA‐6020Austria

**Keywords:** biodiversity, distribution, ecology, epitypification, *Klebsormidium*, molecular phylogeny, morphological characters, phenotypic plasticity

## Abstract

Forty *Klebsormidium* strains isolated from soil crusts of mountain regions (Alps, 600–3,000 m elevation) were analyzed. The molecular phylogeny (internal transcribed spacer rDNA sequences) showed that these strains belong to clades B/C, D, E, and F. Seven main (*K. flaccidum*,* K. elegans*,* K. crenulatum*,* K. dissectum*,* K. nitens*,* K. subtile,* and *K. fluitans*) and four transitional morphotypes (*K*. cf. *flaccidum*,* K*. cf. *nitens*,* K*. cf. *subtile,* and *K*. cf. *fluitans*) were identified. Most strains belong to clade E, which includes isolates that prefer humid conditions. One representative of the xerophytic lineage (clade F) as well as few isolates characteristic of temperate conditions (clades B/C, D) were found. Most strains of clade E were isolated from low/middle elevations (<1,800 m above sea level; a.s.l.) in the pine‐forest zone. Strains of clades B/C, D, and F occurred sporadically at higher elevations (1,548–2,843 m a.s.l.), mostly under xerophytic conditions of alpine meadows. Comparison of the alpine *Klebsormidium* assemblage with data from other biogeographic regions indicated similarity with soil crusts/biofilms from terrestrial habitats in mixed forest in Western Europe, North America, and Asia, as well as walls of buildings in Western European cities. The alpine assemblage differed substantially from crusts from granite outcrops and sand dunes in Eastern Europe (Ukraine), and fundamentally from soil crusts in South African drylands. Epitypification of the known species *K. flaccidum*,* K. crenulatum*,* K. subtile*,* K. nitens*,* K. dissectum*,* K. fluitans*,* K. mucosum,* and *K. elegans* is proposed to establish taxonomic names and type material as an aid for practical studies on these algae, as well as for unambiguous identification of alpine strains. New combination *Klebsormidium subtile* (Kützing) Mikhailyuk, Glaser, Holzinger et Karsten comb. nov. is made.

**Abbreviations**
a.s.l.: above sea level
ITS: internal transcribed spacer
ML: maximum likelihood
SAG: Culture Collection of Algae at Göttingen University, Germany

Biological soil crusts are essential elements of extreme terrestrial habitats, where the growth of higher plants is limited by a complex of unfavorable abiotic factors. These communities represent composite micro‐ecosystems that mainly include bacteria, cyanobacteria, algae, fungi, lichens, liverworts, and mosses, in varying proportions depending on the environmental conditions (Belnap and Lange 2001, Büdel 2002). Biological crusts can stabilize soil surfaces by gluing together sand grains and soil particles, thereby acting as the first (pioneer) stage of soil formation before settlement of higher plant propagules. In addition, these cryptogamic crusts form water‐stable aggregates that have important ecological roles in primary production, nutrient cycling, and water retention of soils (Evans and Johansen 1999, Lewis 2007). Biological soil crusts are present in all terrestrial habitats worldwide, where the growth of vascular plants is limited by low water availability and extremes of temperature (Belnap and Lange 2001).

The algal species composition of cryptogamic crusts in different regions is astonishingly similar and typically includes taxa that are adapted to extreme environments, mostly cyanobacteria (West 1990, Sant'Anna and Azevedo 1991, Rosentreter and Belnap 2001). However, some studies have indicated that the climatic conditions of some regions influence the composition of crusts. Biological soil crusts from deserts of North America (temperate zone) include many green algae (Johansen et al. 1982), whereas significant parts of cryptogamic crusts from Antarctica are composed of prasiolacean green algae (Green and Broady 2001). Filamentous streptophycean green algae (*Zygogonium* Kützing and *Klebsormidium* P.C. Silva, Mattox & Blackwell) dominate the soil crusts of temperate Europe (Lukešová and Komárek 1987, Hoppert et al. 2004), while diatoms and conjugating green algae are the main components of soil crusts in the tundra zone (Skuja 1964).

The Alps constitute an extreme habitat for photosynthetic organisms, including terrestrial algae, due to their harsh climatic and environmental conditions: wide seasonal and diurnal temperature fluctuations, occasional frost in summer, strong impact of wind causing drought and abrasion, and rarefied atmosphere, as well as high levels of insolation including intense ultraviolet radiation that increases with elevation (Lütz and Engel 2007). Investigation of algae present in alpine soil crusts is important because of their possible adaptation to withstand the extreme environmental conditions that are typical for mountains, and because of their essential role in alpine terrestrial ecosystems. Nevertheless, data on the composition and ecology of these algal assemblages are sparse in comparison with the knowledge of soil crusts from arid and polar regions (Türk and Gärtner 2001, Karsten and Holzinger 2014).

Investigations of the species composition, distribution, and ecology of algae from alpine soils began in the 1960s (Pitschmann 1963, Reisigl 1964, 1969, Trenkwalder 1975, Vinatzer 1975). The information was completed and summarized by Reisigl (1964) and later by Türk and Gärtner (2001) and Ettl and Gärtner (1995, 2014). Some data on the species composition and characteristics of alpine soil algae are included in the contributions of Gärtner (2004), Tschaikner et al. (2007, 2008) and Tschaikner (2008). As a result, new species of terrestrial algae from different genera (especially *Heterococcus* Chodat, *Myrmecia* Printz, *Leptosira* Borzi, *Botrydiopsis* Borzi, *Trochisciopsis* Vinatzer, *Coelastrella* Chodat, and others) were described, and some data on the ecology and distribution of known taxa were provided (Ettl and Gärtner 2014). Türk and Gärtner (2001) provided information about the species composition of algae of biological soil crusts in the Alps, which contain abundant filamentous streptophycean and xanthophycean algae, along with cyanobacteria.

Members of the filamentous green alga genus *Klebsormidium* (Klebsormidiophyceae, Streptophyta) are one of the essential components of soil crusts. These algae are widely distributed in terrestrial habitats worldwide (Hoffmann 1989, Lokhorst 1996, Rindi et al. 2008, 2011). The reasons for the ability of *Klebsormidium* to survive and develop high biomass under extremely dry, insolated, hot, or cold terrestrial conditions are not completely understood, but more recent publications indicate a high potential for acclimation to fluctuations in water availability, temperature, and solar radiation (Holzinger and Karsten 2013, Karsten and Holzinger 2014, Kitzing et al. 2014). A recent transcriptomic approach revealed that all prerequisites for living in a terrestrial habitat (e.g., ROS protection mechanisms, and up‐regulation of enzymes involved in the biosynthesis of the raffinose family of oligosaccharides for osmotic protection) are present in *Klebsormidium crenulatum* (Kützing) Lokhorst (Holzinger et al. 2014). The presence of these prerequisites was further supported by a genome‐sequencing study of *Klebsormidium flaccidum* (Kützing) P.C. Silva, Mattox & W.H. Blackwell (Hori et al. 2014). However, the taxonomy of *Klebsormidium* is problematic because of high morphological uniformity and plasticity as well as, probably, a high degree of hidden cryptic diversity (Rindi et al. 2008, 2011, Škaloud and Rindi 2013). Despite the many investigations on morphology, ontogeny, ultrastructure, and phylogeny of *Klebsormidium* (Stewart and Mattox 1975, Lokhorst and Star 1985, Lokhorst 1996, Škaloud 2006, Rindi et al. 2008, 2011, Sluiman et al. 2008, Škaloud and Rindi 2013, Škaloud et al. 2014, Ryšánek et al. 2015 and references therein), unambiguous identification of species in the genus remains difficult. Species delimitation within *Klebsormidium* and even the phylogenetic position of the type species, *K. flaccidum*, are still under debate (Rindi et al. 2011, Škaloud and Rindi 2013, Škaloud et al. 2014). The type material of most of the known *Klebsormidium* species is represented by herbarium sheets (Lokhorst 1996, Rindi et al. 2011), and it is urgently necessary to provide epitypification and designation of the various taxa based on algal strains that are deposited and accessible in culture collections.

Our investigation is part of a broader study on the ecology and ecophysiological performance of *Klebsormidium* as a component of alpine biological soil crusts of the Tyrolean Alps (Karsten et al. 2010, 2013, Holzinger et al. 2011, Kaplan et al. 2012, Karsten and Holzinger 2012, 2014, Kitzing et al. 2014), highlighting its biodiversity using an integrative approach. We isolated strains of *Klebsormidium* from biological soil crusts collected in mountain regions at different elevations (Tyrolean Alps, Austria and Italy, between 600 and 3,000 m a.s.l.), to undertake morphological identifications, to evaluate their genetic diversity, and finally to correlate the biodiversity with the elevational and ecological/biogeographic distributions. Another goal was the epitypification of some well‐described *Klebsormidium* species, to link names to type material, as an improved baseline for future taxonomic studies on original and newly isolated material.

## Material and Methods

### Collection sites, strain isolation and culture conditions

Most of the 40 strains of *Klebsormidium* were isolated from samples of the top 5 mm of alpine biological soil crust collected at different locations in Tyrol (Austria, Italy) during spring 2009; a few samples were collected as biofilms covering rock surfaces or artificial stone substrates. Two strains were isolated in 2007, and five isolates were provided by Prof. Georg Gärtner, University of Innsbruck, Austria. The strain number, origin, and habitat of all the *Klebsormidium* isolates are provided in Table S1 in the Supporting Information.

*Klebsormidium* from the field samples were purified and established as unialgal cultures by the procedure of Tschaikner (2008). All *Klebsormidium* cultures were cultured on solid (1.5% agar) and liquid modified Bold's Basal Medium (Starr and Zeikus 1993) and kept at 20°C and 30–35 μmol photons · m^−2^ · s^−1^ under a light:dark cycle of 16:8 L:D. Osram Daylight Lumilux Cool White lamps (L36W/840; Osram, Munich, Germany) were used as light sources. The cultures are kept in duplicates in the culture collections of the University of Innsbruck, Institute of Botany, Functional Plant Biology and at the University of Rostock, Institute of Biological Sciences, Applied Ecology and Phycology.

### Other *Klebsormidium* strains involved in the investigation

Epitypification of known *Klebsormidium* species was undertaken to link names to their respective type specimens, thus allowing consistent species identification of strains isolated from alpine soil crusts. Eight strains from the Sammlung von Algenkulturen, University of Göttingen, Germany (SAG: Friedl and Lorenz 2012, www.epsag.uni-goettingen.de) were used for comparison with the alpine isolates. Comprehensive information on these strains was previously presented by Rindi et al. (2011).

### Light microscopy and morphological characterization

Young (2‐ to 3‐week old) and old (2‐ to 3‐month old) cultures of all *Klebsormidium* strains were characterized morphologically using an Olympus IX70 light microscope (Olympus Europa Holding, Hamburg, Germany) with Nomarski differential interference optics. Filament morphology was documented with a ColorView II camera (Soft Imaging System GmbH, Münster, Germany) using the imaging software analySIS (Soft Imaging System GmbH). The identification keys of Starmach (1972), Moshkova (1979), Ettl and Gärtner (1995), Hindák (1996) and Lokhorst (1996) were used to identify the taxa prior to the morphological studies. Filament and cell shape and size, morphology of chloroplasts and pyrenoids, presence of H‐like fragments of cell wall and mucilage, growth habit on solid and in liquid medium, as well as modifications of all these characters during the life cycle were documented.

### DNA isolation, PCR, sequencing, and phylogenetic analyses of the *Klebsormidium* strains

**Figure 1:**
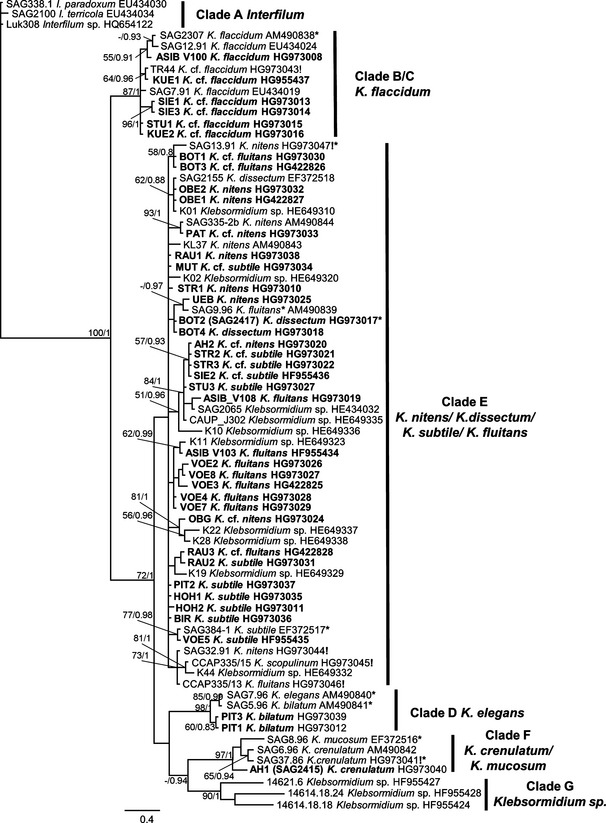
Molecular phylogeny (unrooted) of *Interfilum* (*I*.) and *Klebsormidium* (*K*.) based on ITS‐1 and ITS‐2 rDNA sequence comparisons. Phylogenetic tree was inferred by Bayesian method with Bayesian Posterior Probabilities (PP) and maximum likelihood (ML) bootstrap support (BP) indicated at nodes. From left to right, support values correspond to Bayesian PP and ML BP; BP values lower than 50% and PP lower than 0.8 not shown. Strains marked in bold are sequences of *Klebsormidium* strains from alpine soil crusts. Strains marked with asterisk (*) are proposed as epitypes. Strains marked with exclamation mark (!) are resubmissions of corrected or completed sequences previously published by Rindi et al. (2011). Clade designations follow Rindi et al. (2011).

Genomic DNA was extracted using the DNeasy Plant Mini Kit (Qiagen GmbH, Hilden, Germany). Internal transcribed spacer (ITS) rDNA was amplified in a thermocycler (T gradient Thermoblock, Biometra, Germany) according to Luo et al. (2006) using the Taq PCR Mastermix Kit (Qiagen GmbH) with the primers (EAF3 and ITS055R) published by Marin et al. (2003); PCR products were purified using the Qiagen PCR purification kit (Qiagen GmbH), following the instructions provided by the manufacturer; purified PCR products were sequenced with an ABI 3730 sequencer using the primers 1400F, ITS2F, GF, and GR (Marin et al. 2003, Pröschold et al. 2005). Nucleotide sequences were deposited in GenBank under the accession numbers given in Figure 1 and Table S1. Sequences of strains marked with an exclamation point in Figure 1 were previously published by Rindi et al. (2011), but without 5.8S rDNA. These sequences were completed or corrected by one of us (TM) and were resubmitted to GenBank. The new accession numbers of these sequences are given in Figure 1.

Forty‐nine sequences of *Klebsormidium* and *Interfilum* Chodat strains were used for comparison with strains from alpine soil crusts. These sequences were published by Sluiman et al. (2008), Rindi et al. (2008), and Škaloud and Rindi (2013). Multiple alignments of the newly determined ITS1 and ITS2 rDNA sequences and other sequences selected from the GenBank databases were made using ClustalW and then corrected manually using Bioedit software (Hall 1999). The resulting alignments of the 89 *Klebsormidium* and *Interfilum* strains were a concatenated data set (611 bp) of ITS‐1 (365 bp) and ITS‐2 (246 bp) rDNA sequences according to other researchers working on these genera (Rindi et al. 2008, 2011, Škaloud and Rindi 2013).

To determine the evolutionary model that best fit the data set, the program MEGA version 6 (Tamura et al. 2013) was used. For maximum likelihood (ML), the GTR model with the proportion of invariable sites (I), and the gamma shape parameter (G) resulted in the lowest Akaike Information Criterion (Akaike 1974). For Bayesian analyses, GTR+G had the lowest Bayesian Information Criterion.

The unrooted phylogenetic tree was constructed in MrBayes 3.2.2 (Huelsenbeck and Ronquist 2001, Ronquist and Huelsenbeck 2003) using the GTR+G model with 5,000,000 generations. Two runs of four Monte Carlo Markov Chains were calculated simultaneously, with trees sampled every 500 generations. Split frequency between the runs was below 0.01 at the end of the calculation. The trees sampled before the likelihood scores reached saturation were discarded afterward. The robustness of the tree topology was confirmed by ML (GTR+I+G) performed in GARLI 2.0 (March 2011), and bootstrap support was calculated with 1,000 replicates.

### Statistical analyses

Statistical analyses were done in R software (Version 3.1, R Development Core Team 2009). To visualize the dissimilarities in the composition of *Klebsormidium* clades between different habitats, the non‐metric multidimensional scaling (nMDS) plot was calculated based on the Bray–Curtis dissimilarity index (Bray and Curtis 1957). Goodness of fit was estimated based on the threshold recommended by Clarke and Ainsworth (1993). The distribution patterns of *Klebsormidium* clades along the elevation gradient were visualized by boxplots, also calculated with R software.

## Results

Eleven distinct morphotypes were identified among the alpine *Klebsormidium* strains studied. Seven morphotypes represented known *Klebsormidium* species, according to their morphology: *K. flaccidum K. elegans* Lokhorst, *K. crenulatum*,* K. dissectum* (F. Gay) H. Ettl & G. Gärtner, *K. nitens* (Kützing) Lokhorst, *K. subtile* (Kützing) Tracanna ex Tell and *K. fluitans* (F. Gay) Lokhorst. Four morphotypes were impossible to identify unambiguously with reference to known species, because they were transitional morphological forms between described species. These isolates were identified as follows: *K*. cf. *flaccidum* (transitional morphotype between *K. flaccidum* and *K. dissectum*), *K*. cf. *nitens* (*K. nitens* and *K. dissectum*), *K*. cf. *subtile* (*K. nitens* and *K. subtile*), and *K*. cf. *fluitans* (*K. subtile* and *K. fluitans*).

**Figure 2:**
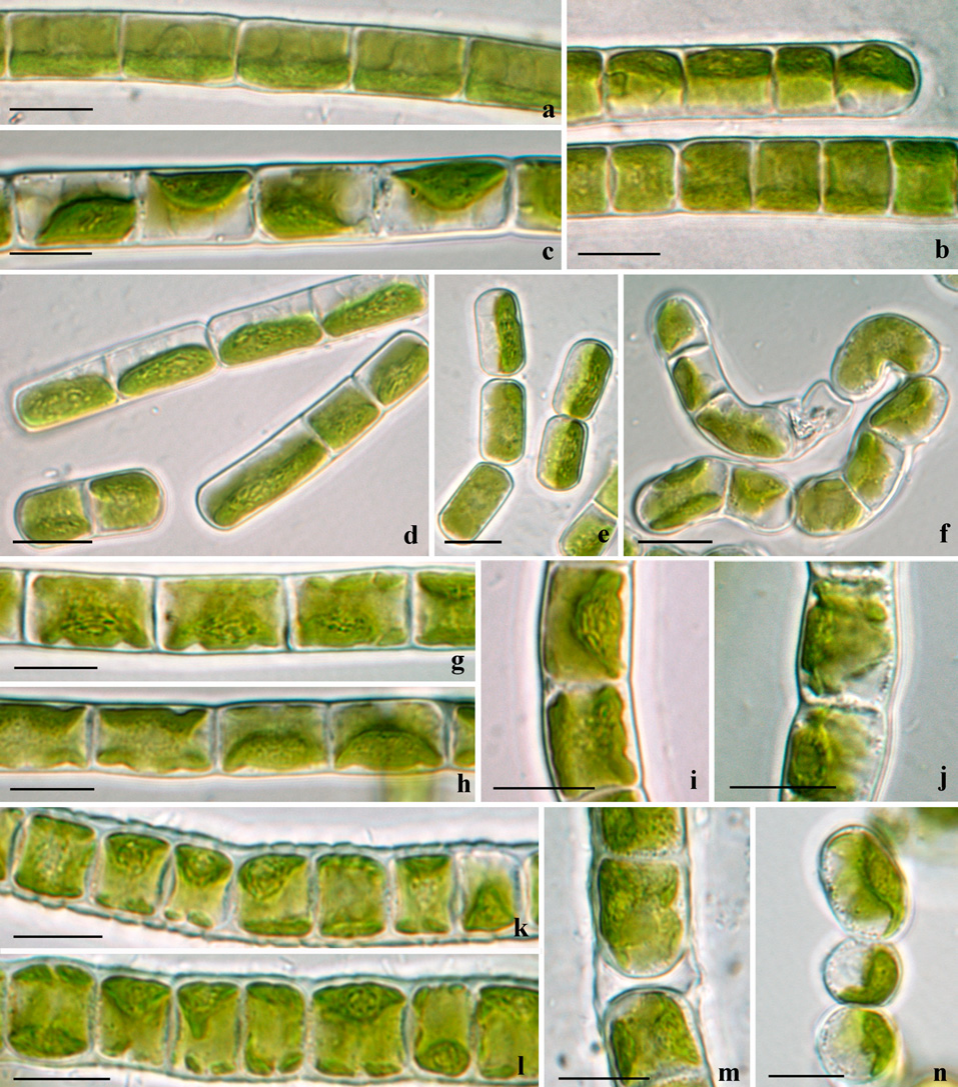
Morphotypes of *Klebsormidium* from alpine soil crusts: (a–c) *K. flaccidum* (ASIB V100), (d–f) *K*. cf. *flaccidum* (KUE1), (g–j) *K. elegans* (PIT3), (k–n) *K. crenulatum* (SAG 2415). (a, b, d, e, g, i, m) Filaments of young (2–3 weeks old), and (c, f, h, g–l, n) filaments of old (2–3 months old) cultures; scale bars: 10 μm.

**Figure 3:**
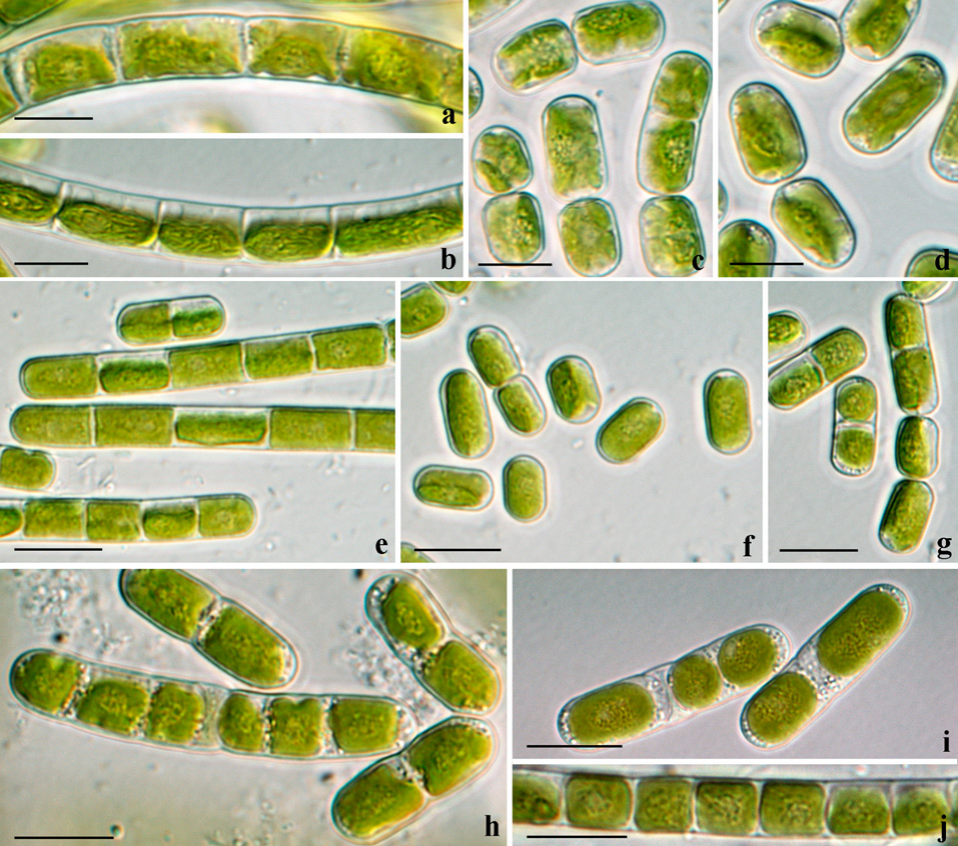
Morphotypes of *Klebsormidium* from alpine soil crusts: (a–d) *K. dissectum* (SAG 2417), (e–g) *K. nitens* (OBE1 (e), STR1 (f, g), (h–j) *K*. cf. *nitens* (PAT (h), AH 2(SAG 2416) (i), OBG (j)). (b, e, f, j) Filaments of young (2–3 weeks old), and (a, c, d, g–i) filaments of old (2–3 months old) cultures; scale bars: 10 μm.

**Figure 4:**
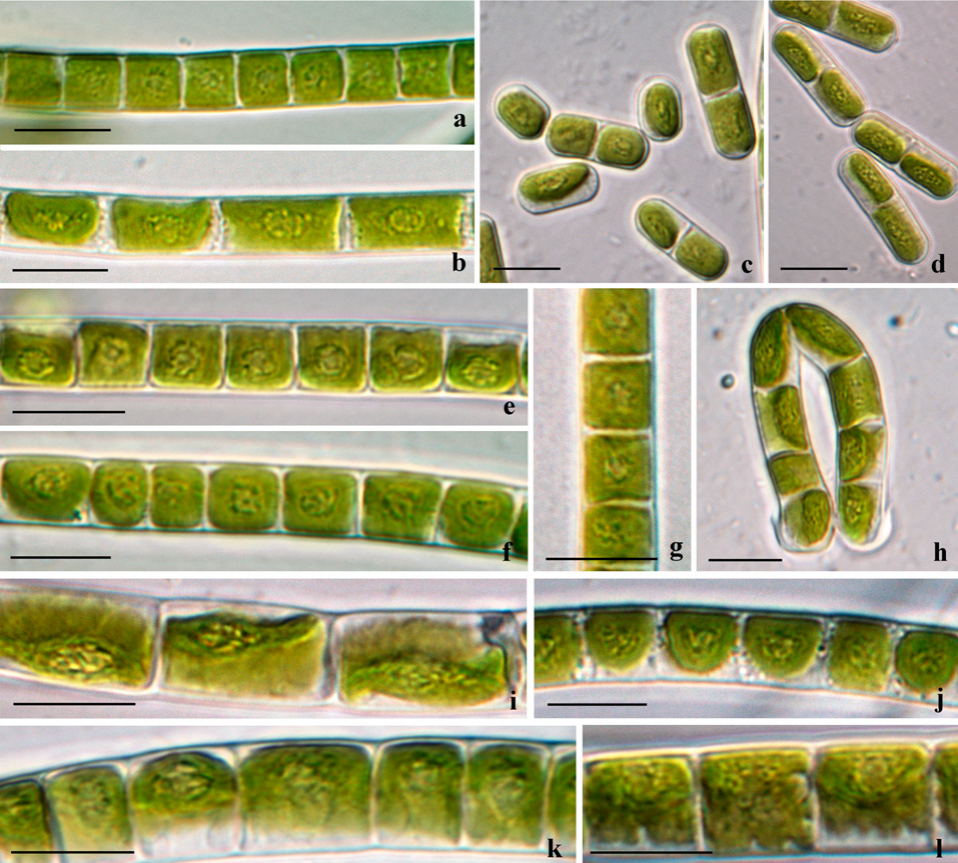
Morphotypes of *Klebsormidium* from alpine soil crusts: (a–d) *K*. cf. *subtile* (STR2 (a), SIE2 (b–d), (e–h) *K. subtile* (VOE5 (e), HOH2 (f, g), PIT 2 (h)), (i, j) *K*. cf. *fluitans* (BOT3), (k, l) *K. fluitans* (VOE2 (k), ASIB V103 (l). (a, c–e, j–i, k) Filaments of young (2–3 weeks old) and (b, f, j, l) filaments of old (2–3 months old) cultures; scale bars: 10 μm.

*Klebsormidium* is characterized by a high level of morphological plasticity, and hence we often found different morphotypes over the course of repeated observations on the same strain, reflecting different culture ages and developmental stages. The representatives with thin or medium‐sized filaments had in general a high level of variability. This variation in what are considered informative taxonomic characters, such as cell length and filament width and the degree of its disintegration, influenced the general appearance of *Klebsormidium* filaments. Descriptions and images of morphotypes of alpine *Klebsormidium* strains are presented in Table 1 and Figures 2, 3, 4.

**Table 1:** Description of *Klebsormidium* morphotypes from alpine soil crusts collected at different elevations

Morphospecies	Strain	Elevation m a.s.l.	Description
*K. flaccidum*	ASIB V100	2,363	Filaments long, (7.8)8.1–8.5(9.3) × (7.3)10.7–14.3(17.8) μm, with tendency to disintegration, not or slightly constricted; cells cylindrical, (1)1.3–1.5(1.8) times as long as wide; cell wall moderately thickened; H‐pieces present rarely; chloroplast covers 1/2–2/3 of the cell inner surface, with smooth margins, pyrenoid large, surrounded by several layers of starch grains. In liquid media forming superficial hydrorepellent layer and submerged tufts; on agar forming undulating colonies
*K*. cf. *flaccidum*	SIE1	1,548	Similar to *K. flaccidum*, but with much stronger tendency to disintegration, especially in old culture, cells (6.5)7–8.3(11.7) × (4.0)5.7–12.1(13.2) μm, chloroplast usually with crenulated or irregularly dissected margins, appears to be intermediate between *K. flaccidum* and *K. dissectum* morphospecies
	SIE3	1,548	
	KUE1^a^	2,435	
	KUE2	2,435	
	STU1	2,866	
*K. elegans*	PIT1	2,843	Filaments long, robust, (8)8.5–9.5(9.8) × (5)7.3–11.8(16.7) μm, sometimes growing in rope‐like aggregates; cells cylindrical to barrel‐shaped, (1.1)1.2–1.7 times as long as wide; cell wall moderately thickened; H‐pieces present, prominent; chloroplast covering half to 3/4 of the cell inner surface, with a median incision in the margin, dissected in four or more lobes; pyrenoid large, surrounded by several layers of starch grains. In liquid media forming submerged tufts; on agar forming rough undulating colonies
	PIT3	2,843	
*K. crenulatum*	AH1 (SAG 2415)^b^	2,350	Filaments long, strong, thick, (9.2)10–11.5 × (6.4)7.1–12.9(16.8) μm wide, sometimes growing in rope‐like aggregates; cells cylindrical, becoming barrel‐shaped and sub quadrate in old filaments, 0.5–1(1.5) times as long as wide; cell doublets occasionally present; cell wall initially thin, becoming thick and corrugated in old filaments; H‐pieces common, prominent; chloroplast girdle‐shaped, almost ring‐like, covers most of the cell inner surface, with longitudinal margins smooth or slightly lobed; pyrenoid large, surrounded by several layers of starch grains. In liquid media forming only submerged tufts, on agar forming rough, undulating colonies
*K. dissectum*	BOT2 (SAG 2417)^c^	609	Filaments moderately long, but easily disintegrated, especially in mature and old cultures, (6)6.4–8.6(9.3) × (5.9)8.9–15.5(16.7) μm, slightly or distinctly constricted; cells cylindrical, often slightly swollen, in unicell stage ellipsoid or ovoid, (1.1)1.5–1.8(2.5) times as long as wide; cell wall moderately thickened; H‐pieces usually absent; chloroplast covers 1/2–2/3 of the cell inner surface, with margins crenulated or irregularly dissected; pyrenoid somewhat large, surrounded by several layers of starch grains. In liquid media forming superficial hydrorepellent layer and submerged tufts; on agar forming homogeneous colonies with crenulate margins
	BOT4	609	
*K. nitens*	OBE1^d^	1,046	Filaments short, thin, (4.5)5–5.9(6.7) × (6.3)7.2–13.6(19.5) μm; easily disintegrated to unicells; cells cylindrical, constricted, 1.3–1.5(3) times as long as wide; cell wall thin; H‐pieces usually absent; chloroplast covers 1/2–2/3 of the cell inner surface, with delicately lobed margins; pyrenoid small, surrounded by a layer of starch grains. In liquid culture forming submerged tufts and superficial layer; on agar forming smooth colonies
	OBE2	1,046	
	RAU1	1,074	
	STR1	1,280	
	UEB	1,680	
*K*. cf. *nitens*	PAT	2,145	Similar to *K. nitens*, but filaments thin to medium width, (5.3)5.6–6.8(7.8) × (6.3)7.2–10.9(13.6) μm; sometimes unicellular stage with ellipsoid cells and lobed chloroplast resembling *Interfilum* species
	AH2 (SAG 2416)^e^	2,350	
	OBG	2,350	
*K. subtile*	STU3	649	Filaments long, with some tendency to fragmentation, medium width, in young culture cells are long and cylindrical, in mature and old cultures the cells become isodiametric (length/width—(0.8)1–1.3(1.8)), (5.4)6.0–6.8(7.6) × (4.7)5.8–10.3(13.1) μm, filaments are slightly bead‐like, constricted near cross walls, H‐like pieces sometimes present; chloroplast covers 2/3 of the cell inner surface, with smooth or waved margins; the pyrenoid is small, round, compact, surrounded by a layer of starch grains. In liquid culture forming submerged tufts and superficial layer; on agar forming smooth colonies
	RAU2	1,074	
	BIR	1,953	
	HOH1	2,207	
	HOH2	2,207	
	PIT2	2,843	
	VOE5^f^	2,866	
*K*. cf. *subtile*	STR2	1,280	Similar to *K. subtile*, but filaments often fragmented and usually in mature culture have long, filaments and unicells, from thin to medium in width, (5.4)5.8–6.8(7.2) × (4.0)5.6–10.8(11.9) μm
	STR3	1,280	
	SIE2^g^	1,548	
	MUT	2,650	
*K. fluitans*	VOE2	649	Filaments long, strong, sometimes with tendency to disintegration, (6.5)7.5–8.8(10.7) × (6.6)7.1–11.5 (17.4) μm; cells cylindrical to isodiametric, slightly swollen, (0.6)0.9–1.4 times as long as wide; cell wall of medium thickness; H‐pieces present; chloroplast covers 1/2–3/4 of the cell inner surface, with margins smooth or delicately crenulated; pyrenoid medium‐sized, surrounded by several layers of starch grains. In liquid culture submerged tufts present; on agar forming growths with rough surface
	VOE3^d^	649	
	VOE4	649	
	VOE7	649	
	VOE8	649	
	ASIB V103^h^	2,363	
	ASIB V108	2,363	
*K*. cf. *fluitans*	BOT1	609	Similar to *K. fluitans*, but in culture abundant thick filaments often present together with thin filaments similar to *K. subtile* morphotype, cell width (5.4)6.4–7.8(8.8) μm
	BOT3^d^	609	
	RAU3^d^	1,074	

a Strain KUE1 was previously identified as transitional morphotype between *K. flaccidum* and *K. dissectum*, based on morphological characters (Karsten et al. 2013).

b Strain AH1 (SAG 2415) was previously identified as *K. crenulatum*, based on morphological characters (Karsten et al. 2010, Holzinger et al. 2011) and *rbc*L phylogeny (Kaplan et al. 2012).

c Strain BOT2 (SAG 2417) was previously identified as *K. nitens*, based on *rbc*L phylogeny (Kaplan et al. 2012).

d Strains OBE1, VOE3, BOT3 and RAU3 were previously identified as *K. fluitans*, based on ITS phylogeny (Kitzing et al. 2014).

e Strain AH2 (SAG 2416) was previously identified as *K. dissectum*, based on morphological characters (Karsten and Holzinger 2012).

f Strain VOE5 was previously identified as a transitional morphotype between *K. subtile* and *K. subtilissimum*, based on morphological characters (Karsten et al. 2013).

g Strain SIE2 was previously identified as a transitional morphotype between *K. nitens* and *K. dissectum*, based on morphological characters (Karsten et al. 2013).

h Strain ASIB V103 was previously identified as *K. fluitans*, based on morphological characters (Karsten et al. 2013) and ITS phylogeny (Kitzing et al. 2014).

The Bayesian phylogenetic tree of ITS rDNA sequences is presented in Figure 1. Six previously described clades are shown on the tree: A, B/C, D, E, F, and G. The ITS phylogeny did not clearly differentiate between clades B and C. Clade E, which included the majority of strains, had weak statistical support and limited resolution of some subclades.

**Figure 5:**
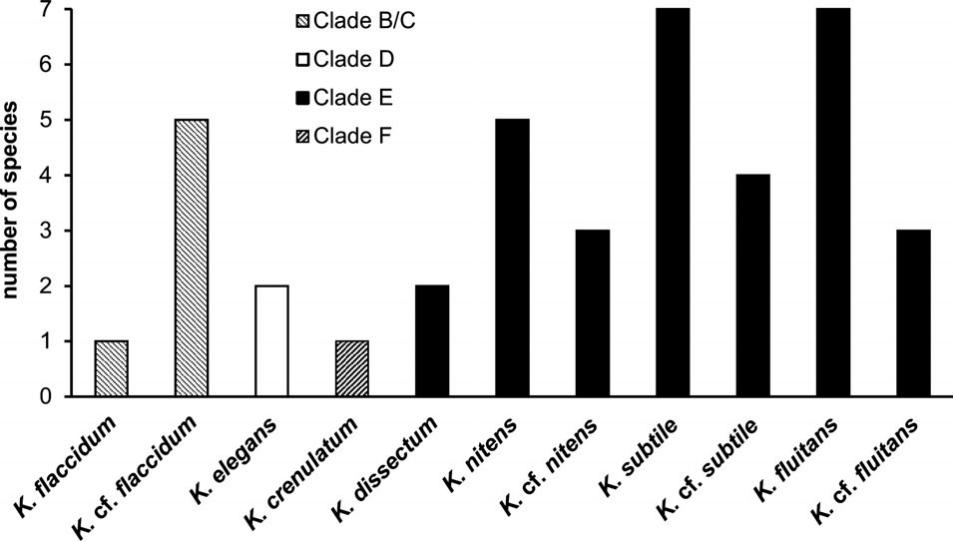
Total number of *Klebsormidium* strains found in alpine soil crusts, assigned to phylogenetic clades and morphotypes.

Forty of the strains from alpine soil crusts were distributed among the main phylogenetic lineages of *Klebsormidium*: clades B/C, D, E, and F. An exception was clade G, which is composed mostly of strains isolated from arid regions. The majority of alpine strains (31, 77.5%) were included in clade E, which contains the largest number of taxa (Fig. 5): *K. dissectum*,* K. nitens*,* K*. cf. *nitens*,* K. subtile*,* K*. cf. *subtile*,* K. fluitans,* and *K*. cf. *fluitans*. Many fewer strains were distributed among clades B/C (6 strains, 15.0%), D (2 strains, 5.0%) and F (1 strain, 2.5%). Clade B/C united two morphotypes: *K. flaccidum* and *K*. cf. *flaccidum*. Clades D and F included one morphotype each: *K. elegans* and *K. crenulatum*, respectively.

**Figure 6:**
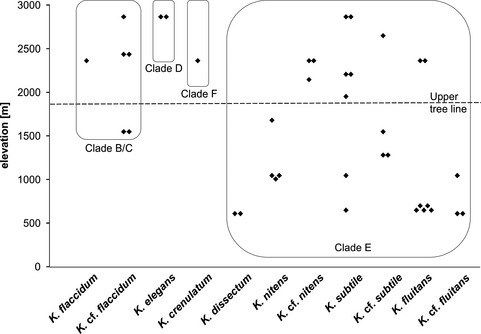
Distribution of alpine *Klebsormidium* strains along elevation gradient and among phylogenetic clades and morphotypes.

The distribution of *Klebsormidium* morphotypes and lineages along the elevation gradient showed some clustering (Fig. 6). Strains from the largest clade E were distributed evenly over the elevations sampled. However, a closer look revealed that strains of the *K. dissectum* and *K*. cf. *fluitans* morphotypes were collected only at lower elevations, whereas *K. nitens* occurred at middle elevations, and morphotype *K*. cf. *nitens* at high elevations. Four of six members of clade B/C, along with all strains of clades D and F, were collected at high elevations (Fig. 6).

## Discussion

### Distribution of alpine *Klebsormidium* strains among different phylogenetic clades and along elevation gradients

The phylogeny presented in Figure 1 corresponds well with the ITS phylogeny of *Klebsormidium* and *Interfilum* published by Rindi et al. (2011) and with the ITS‐*rbc*L phylogeny from more recent publications (Škaloud et al. 2014, Ryšánek et al. 2015). However, clade A (*Interfilum*), which usually appears as a sister group to clade B/C, is distant from other *Klebsormidium* clades (B/C, D, E, F, and G). As the largest number of strains isolated from the alpine habitats were representatives of clade E it formed the base of biodiversity for the alpine *Klebsormidium* strains (77.5%). In addition, this group also united the largest number of *Klebsormidium* morphotypes (Fig. 5). Clade E is the most common group worldwide, and is typical for terrestrial habitats of Europe, North America, and Asia (Ryšánek et al. 2015). Alpine strains were found in all known *Klebsormidium* lineages, with the exception of clade G. This clade consists mostly of strains isolated from arid regions such as in Africa (Rindi et al. 2011), and hence did not contribute to the *Klebsormidium* biodiversity of the Alps. More recently, however, several strains of *Klebsormidium* from acidic soils in Europe were added to clade G (Škaloud et al. 2014).

**Figure 7:**
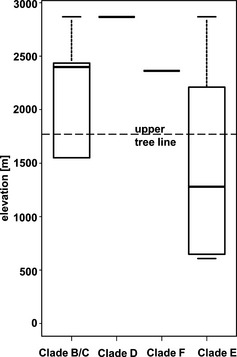
Boxplots showing median, 25%–75% percentiles and range of distribution of *Klebsormidium* phylogenetic clades in alpine soil crusts along elevation gradient.

Although the number of samples is too small for a proper correlation analysis, at least some conspicuous trends among the *Klebsormidium* morphotypes/clades and their elevational distributions were apparent (Figs. 6 and 7). Representatives of clades B/C, D, and F were collected mainly from soil crusts at high elevations near the pine‐forest line and above on alpine meadows and in the nival belt. In contrast, strains from clade E were mostly found at low and middle elevations in the forest zone. In total, 20 *Klebsormidium* strains from clade E (64.5%) were isolated <1,800 m a.s.l. within the zone of pine forests, and 11 strains (35.5%) were found above this level. Therefore, despite their morphological and ecological plasticity, strains of clade E in general may be more typical of humid and shaded habitats globally (e.g., Škaloud and Rindi 2013), such as pine forest. Strains of clades B/C, D, and F appear to be more adapted to xerophytic habitats characteristic of higher elevations exposed to greater amounts of solar radiation.

### Comparing distribution patterns of *Klebsormidium* phylogenetic clades from alpine soil crusts to terrestrial habitats from other regions

We compared our biodiversity data on the alpine *Klebsormidium* isolates with those from other regions to verify our proposed ecological preferences of *Klebsormidium* strains from different phylogenetic lineages, as well as to reveal species composition patterns in terrestrial habitats of the Alps. We selected studies that reflected the most comprehensive *Klebsormidium* biodiversity of some terrestrial habitats, using an integrative approach. Although the monograph of Kostikov et al. (2001) contains only morphological data, it was very helpful because of the detailed descriptions of easily identifiable species (*K. crenulatum* / *K. mucosum* (J.B. Peteresen) Lokhorst complex).

**Figure 8:**
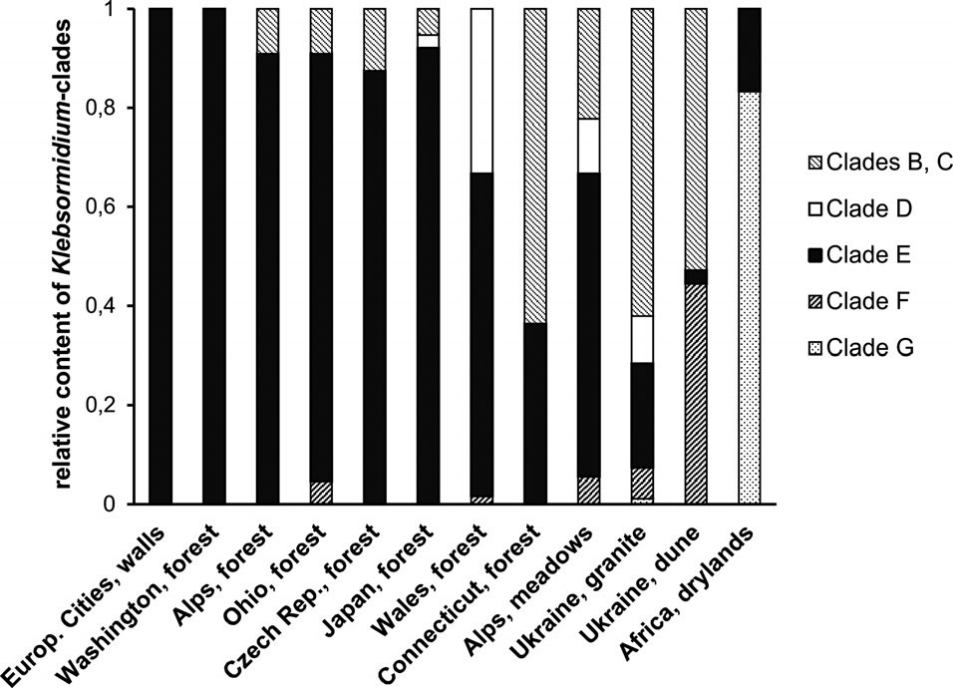
Comparison of distribution pattern of phylogenetic clades within *Klebsormidium* collected from alpine soil crusts (“Alps, forest” and “Alps, meadows”) and other sites. “Europ. cities, walls” refers to algal crusts from building walls of Western European cities (Rindi et al. 2008); “Washington, forest,” “Ohio, forest,” “Connecticut, forest,” “Czech Rep., forest,” “Wales, forest” and “Japan, forest” are terrestrial habitats under mixed forest of northern temperate zones of United StatesA, Western Europe and Japan (Ryšánek et al. 2015); “Ukraine, granite” stands for algal crusts from granite outcrops in Ukraine (Mikhailyuk et al. 2011); “Ukraine, dunes” refers to soil crusts from dunes along Dnieper River Dnipro sand dunes in Ukraine (Kostikov et al. 2001); “Africa, drylands” to soil crusts from South African drylands (Büdel et al. 2009, Rindi et al. 2011).

The results of our analysis are presented in Figure 8. Differences in species composition of *Klebsormidium* in alpine soil crusts appear related more to different habitats (forest or meadows) than to elevations (see Fig. 7). Therefore the assemblage of *Klebsormidium* from alpine soil crust was divided into two groups, according to their site of collection in either forest or meadows. The species composition of *Klebsormidium* from different terrestrial habitats (soil, stones, and tree bark) under cover of mixed forests in Washington and Ohio (United States), Czech Republic and Wales (Western Europe), and Japan (Asia) is similar to that of alpine soil crusts from the forest zone, with a conspicuous dominance of representatives of clade E. Algal biofilms growing on building walls in different cities of Western Europe are similar as well, because they consist solely of clade E strains. Although *Klebsormidium* communities from alpine meadows are more diverse, those close to forest ecosystems include a high number of strains from clade E. This phylogenetic lineage includes several groups of freshwater *Klebsormidium* strains (clade E1 according to Rindi et al. 2011, or clades 5, 6, 8, 9, 13, and 14 according to Škaloud and Rindi 2013). Some ecophysiological data on E clade strains indicate a high sensitivity to desiccation (Karsten and Rindi 2010, Karsten and Holzinger 2012). Therefore, it is reasonable to assume that this group of *Klebsormidium* species is, in general, adapted to more hydrophilic habitats, because it is widely distributed in humid and shaded habitats of Western Europe, North America, and Asia, as well as at middle elevations in the Alps, especially in forest belts where adequate humidity is always available.

Biofilms from granite outcrops of steppe slopes and from sand dunes of the Ukraine showed a different composition of *Klebsormidium* species: a predominance of strains from clade B/C, with a strong contribution of clade F in the assemblage (Fig. 8; Kostikov et al. 2001, Mikhailyuk et al. 2011). Members of clade F undoubtedly represent a xerophytic adapted lineage of *Klebsormidium*, according to their characteristic morphological and ultrastructural features (thick cell walls, narrow cells, and a tendency of filaments to fold in braids, which aids in self‐protection by preventing excessive water evaporation) along with a high desiccation tolerance (Karsten et al. 2010, Holzinger et al. 2011, Kaplan et al. 2012). It is possible that representatives of clade B/C are more xerophytic than strains of clade E, regardless of the morphological similarity of this genetically separated lineage. Particularly the more xerophytic clades of *Klebsormidium* (B/C, F) have a wider distribution in terrestrial habitats of open landscapes of the Ukraine, due to the more continental (drier) climate and more insolated conditions on steppe slopes and sand dunes. Recently, it was reported that clade B/C does not include acidophilic strains, which were found in all other lineages of *Klebsormidium* (Škaloud et al. 2014).

The soil crusts from savannas and deserts of South Africa have a unique assemblage of *Klebsormidium* species that may reflect the specific climatic conditions of these habitats (Büdel et al. 2009, Rindi et al. 2011). Most of these strains of *Klebsormidium* belong to the unique and recently discovered phylogenetic lineage clade G. This lineage is xerophytic as well, as its representatives have morphological similarities to species of clade F (thick cell walls and narrow cells, and strongly curved filaments arranged in ball‐like aggregations and cluster‐like colonies). Strains of clade E, typical of rather humid conditions, are found in the savannas and deserts of South Africa only sporadically.

**Figure 9:**
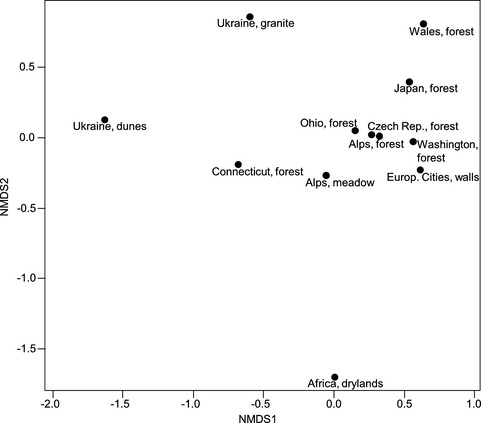
nMDS plot based on Bray–Curtis dissimilarity index visualizes differences in composition of *Klebsormidium* clades, based on absolute numbers in alpine region and other terrestrial habitats. For explanation of symbols, see legend of Figure 8; stress = 0.06.

The nMDS plot (Fig. 9) clearly confirms the expected similarity of the *Klebsormidium* species composition among alpine soil crusts from forest habitats of Western Europe, North America, and Asia (stress = 0.06). The *Klebsormidium* assemblages of alpine soil crusts from meadows and from forests in the U.S. occupy a transitional position between those of alpine soil crusts in Western Europe, North America, and Asia, and those of open landscapes in Eastern Europe. The species composition of African *Klebsormidium* points to a very high dissimilarity compared with all other habitats, due to the presence of members of clade G (Fig. 9).

*Klebsormidium* is a cosmopolitan genus, and its members seem to be easily dispersed via air transport (Hoffmann 1989, Ettl and Gärtner 1995, 2014, Lokhorst 1996, Rindi et al. 2011). Ecological differentiation has been described for some genetic lineages and clades (Rindi et al. 2011, Škaloud and Rindi 2013), showing that not geographic, but rather ecological factors determine the distribution of members of the genus, because no endemic lineages could be detected (Ryšánek et al. 2015). However, it appears that ecological and geographic factors are interdependent, as climatic peculiarities determine the characteristics of a habitat. Although geographic barriers do not affect the distribution of microscopic organisms (Finlay et al. 1996, Finlay 2002), the geographic position determines environmental conditions. Our analysis indicates the influence of both parameters (geographic and ecological) on the distribution of *Klebsormidium* species in soil crusts. The composition of *Klebsormidium* assemblages from shaded or open habitats (e.g., forests or steppes, savannas) in different geographic regions exhibits similarities due to the influence of comparable microclimatic conditions. Despite statements that the species composition of organisms forming soil crusts (lichens, mosses, and algae) in different regions is similar because of adaptation to extreme environments (West 1990, Sant'Anna and Azevedo 1991, Rosentreter and Belnap 2001, Türk and Gärtner 2001), our data showed that a key genus of soil crusts, *Klebsormidium*, contains different genetic lineages with different ecological features, depending on the local environmental conditions.

### Identification of alpine strains, problems of species delimitation, morphological plasticity

The phylogenetic analysis of *Klebsormidium* alpine isolates (Fig. 1) showed that only a small number of the described morphotypes clearly corresponded to known taxa. Alpine strains assigned to the *K. flaccidum* and *K. dissectum* morphotypes, along with one strain of the *K. subtile* morphotype showed high similarity to the respective epitypes (see below). Two strains of the morphotypes *K. elegans* and *K. crenulatum* were included in clades D and F, respectively, but each formed a separate subclade. Strains belonging to the morphotypes *K. fluitans*,* K. nitens*, and *K. subtile* (in part) appeared in several subclades of E, far distant phylogenetically from the corresponding epitype strains. Data concerning the polyphyletic position of these morphotypes were partially reported in previous papers (Rindi et al. 2011, Škaloud and Rindi 2013, Škaloud et al. 2014). Some of our alpine strains represented transitional morphotypes (*K*. cf. *flaccidum*,* K*. cf. *nitens*,* K*. cf. *subtile*, and *K*. cf. *fluitans*) that do not clearly correspond to existing morphological species. The strains of the *K*. cf. *flaccidum* morphotype belonged to clade B/C, but formed separate subclades (Fig. 1). Morphotypes of *K*. cf. *nitens*,* K*. cf. *subtile,* and *K*. cf. *fluitans* were all distributed within clade E, in no clear order, but with a gradual transition from one morphotype to another (Fig. 1).

Some alpine strains of *Klebsormidium* were mentioned in other publications, where they were identified based on morphology or ITS/*rbc*L phylogeny (see Table 1). While most identifications could be confirmed, two strains BOT2 (SAG 2417) and OBE1 were different: *K. nitens* as described by Kaplan et al. (2012) represents *K. dissectum*, and *K. fluitans* as described by Kitzing et al. (2014) is a member of *K. nitens*. This misidentification is related to the absence of reference strains for most species of *Klebsormidium*. Identification based purely on molecular data, as in the above papers, without comparison to morphology is problematic. Therefore, a comprehensive taxonomic revision of *Klebsormidium*, combining molecular, morphological, and ecological data is needed.

Recent data of other authors showed the existence of a high number of cryptic *Klebsormidium* species within clade E (Škaloud and Rindi 2013), as determined on the basis of ITS‐*rcb*L phylogeny and some ecological preferences. Sequences of several strains published by these authors are included in our phylogenetic tree. Alpine strains formed separate subclades and corresponded to some cryptic species according to Škaloud and Rindi (2013), but were distributed among other, previously known strains in no clear order (Fig. 1). Therefore, it is difficult to reach a definite conclusion on the taxonomic position of some alpine isolates, or on their ecological preferences, because they are distributed among taxa identified by other authors as cryptic species characteristic of artificial subaerial substrates or freshwater habitats (Škaloud and Rindi 2013). We agree with the statement of these authors that the morphological, genetic, and ecological variability in clade E members is difficult to address because of high plasticity with respect to these parameters.

We compared our data with those of an early publication (Reisigl 1964) on the diversity of *Klebsormidium* from alpine soils, which was later cited by Türk and Gärtner (2001) and Ettl and Gärtner (1995, 2014). Four morphotypes of *Klebsormidium* (Types 1, 2, 3, and 4) referred to *Hormidium flaccidum* (Kützing) A. Brown were found in alpine soils at elevations between 3,457 and 3,739 m a.s.l. (Reisigl 1964). Reisigl used a wide species concept for *H. flaccidum* and mentioned a high morphological plasticity in culture as well as taxonomic problems with the genus. Although a precise evaluation of Reisigl's data is difficult because of the brief descriptions and the absence of illustrations of the four morphotypes, most probably all of these morphotypes belonged to clade E, as they consisted of thin filaments (~5.5 μm diameter). Another species, *K. montanum* (Hansgirg) S.Watanabe, found in soil in South Tyrol, Italy (Ettl and Gärtner 1995, 2014) represents a morphotype very similar to the *K. crenulatum*/*K. mucosum* complex.

### Epitypification of known *Klebsormidium* species

Identification of *Klebsormidium* species remains difficult for several reasons including incomplete species descriptions without illustrations in the 19th century (Kützing 1845, 1849, Rabenhorst 1857, Gay 1891, 1894) and further changing species concepts as a result of the studies of several generations of scientists (Klebs 1896, Mattox 1968, Silva et al. 1972, Starmach 1972, Tell 1976, Moshkova 1979, Ettl and Gärtner 1995, 2014, Hindák 1996, Lokhorst 1996, Novis 2006, Sluiman et al. 2008, Rindi et al. 2011). For example, the type species of *K. flaccidum* was described as *Ulothrix flaccida* Kützing with a short diagnosis where data about filament structure, dimensions and shape of cells, presence of nucleus and locality were provided (Kützing^1849^). Actually, it was described as a green terrestrial alga with filaments of average width in comparison with *U. nitens* Kützing (thinner filaments) and *U. crenulata* Kützing (thicker filaments), both described at the same time. Later much more data were added to the species diagnosis, including chloroplast and pyrenoid structure, presence/absence of H‐like fragments of cell wall, details of asexual reproduction, macroscopic growth habit, ecological features, and distribution (Klebs 1896, Starmach 1972, Moshkova 1979, Ettl and Gärtner 1995, 2014, Hindák 1996, Lokhorst 1996, Sluiman et al. 2008, Rindi et al. 2011). However, even more recent publications exhibit many contradictions concerning the characterization of *Klebsormidium* species. For example, width of filaments of *K. flaccidum* is 5.5–6 μm in Starmach (1972), (5.6) 6.5–7.4 μm in Lokhorst (1996), but much broader in Moshkova (1979), Ettl and Gärtner (1995, 2014), and Hindák (1996) (5–14 μm).

For taxa assigned to *Klebsormidium*, we propose using species' protologues as well as Lokhorst (1996) to designate epitypes. Lokhorst (1996) provided the most comprehensive morphological treatment of *Klebsormidium* species in Western Europe, based primarily on cultured material. His work is valuable because Lokhorst (1996) designated holotypes, lectotypes, or neotypes of all *Klebsormidium* species that he investigated. Unfortunately, Lokhorst's monograph does not refer to any strain numbers, and type material cannot be used to study morphological plasticity and molecular phylogeny (Rindi et al. 2011, Škaloud et al. 2014) or even to identify species (Pröschold and Leliaert 2007, Friedl and Rybalka 2012). According to Article 9.8 of the ICN (McNeill et al. 2012), “An epitype is a specimen or illustration selected to serve as an interpretative type when the holotype, lectotype, or previously designated neotype, or all original material associated with a validly published name, is demonstrably ambiguous and cannot be critically identified for purposes of the precise application of the name to a taxon.” For *Klebsormidium* species, as for many other species of microalgae (e.g., Darienko et al. 2010, Bock et al. 2011, Demchenko et al. 2012, Rybalka et al. 2013), epitypification is necessary to unambiguously link names to sequenced specimens. In most cases, the strains isolated and investigated by Lokhorst (1996) are proposed as epitypes, except for *K. subtile* that he did not treat. For each recognized species, we usually accept Lokhorst's (1996) heterotypic synonyms.

*Klebsormidium flaccidum* (Kützing) Silva et al. (1972). Taxon 21:643.

*Basionym: Ulothrix flaccida* Kützing (1849: 349), Species Algarum.

*Synonyms*: see Lokhorst (1996: 17).

*Type locality*: Stony road in Strasbourg (France).

*Emended description*: Lokhorst (1996) Cryptogamic Studies 5: 17–20, figs. 31–68.

*Lectotype*: L 939.67‐905, annotated as “Strassburg Febr 1846,” leg. A. Braun, designated by Lokhorst (1996).

*Epitype*: Strain SAG 2307 designated here to support the lectotype specified above and the authentic strain of *K. flaccidum* that is permanently preserved in a metabolically inactive state (cryopreserved in liquid nitrogen) in the SAG.

*Comments*:* Klebsormidium flaccidum* has a simple morphological appearance and therefore could be identified as strains of several genetic lineages of *Klebsormidium* (clades B, C, or E; Rindi et al. 2011, Škaloud et al. 2014). The herbarium type material and the incomplete original diagnosis cannot be used to clarify the situation with this species. Therefore, the designation of an epitype specimen based on a subjective choice is the only feasible solution for a reassessment for this species (Rindi et al. 2011, Škaloud et al. 2014). We propose a strain of *K. flaccidum* isolated by Lokhorst and now preserved in the SAG collection (SAG 2307) as the epitype. SAG 2307 was isolated from clayey soil in a field of beets near Niederkruechten (Germany), original number KL 1. It does not conflict with the original description, is ~400 km from the type locality (Kützing 1849), and generally corresponds to the emended description. Minor differences include the slightly wider filaments, 7.8(8.8) μm (in the emended description—(5.6)6.5–7.4 μm). The epitype strain rarely has H‐like fragments of cell walls in agar culture and has a prominent starch envelope composed of several layers of small starch grains surrounding the pyrenoid (the starch envelope of the pyrenoid is visible in Lokhorst's micrographs (Lokhorst 1996, figs. 44, 45), although the description mentions “without distinct starch envelope”).

*Klebsormidium crenulatum* (Kützing) Lokhorst in Lokhorst and Star (1985). *J. Phycol*. 21:474.

*Basionym*:* Hormidium crenulatum* Kützing (1845:193), Phycol.Germ.

*Synonyms*:* Klebsormidium crenulatum* (Kützing) H. Ettl & G. Gärtner nom. inval.; see also Lokhorst 1996 (p. 30).

*Type locality*: On wet and warm wall of a bath house, Padua (Italy).

*Emended description*: Lokhorst (1996). Cryptogamic Studies 5: 30–34, figs. 181–219.

*Lectotype*: L 939.67‐834, annotated as “1/280” *Hormidium crenulatum*, Patavii, leg. G. Meneghini, designated in Lokhorst and Star (1985).

*Epitype*: Strain SAG 37.86 designated here to support the lectotype specified above and the authentic strain of *K. crenulatum* that is permanently preserved in a metabolically inactive state (cryopreserved in liquid nitrogen) in the SAG.

*Comments*: Strain SAG 37.86 was isolated by H. Trenkwalder (original number T 93) from a soil from Brixen, South Tyrol (Italy) and was previously identified as *Ulothrix tenuissima* Kützing. Strain SAG 6.96, isolated by Lokhorst (original number KL 64) is no longer available. The phylogenetic analysis provided by Rindi et al. (2011) showed a close relationship between the two isolates. Strain SAG 37.86 does not conflict with the original description and type locality (Kützing 1845) and generally corresponds well with the emended description, but differs in having slightly thicker filaments, reaching 16.6(17.8) μm (the original description gives the maximum width as 14.0(15.8) μm). The taxonomic combination *Klebsormidium crenulatum* (Kützing) H. Ettl & G. Gärtner (Ettl and Gärtner 1995) is invalid, because of priority rules.

***Klebsormidium subtile*** (Kützing) Mikhailyuk, Glaser, Holzinger et Karsten **comb. nov.**

*Basionym*:* Ulothrix subtilis* Kützing (1845). Phycol.Germ.: 197 pro parte.

*Synonyms*:* Stichococcus subtilis* (Kützing) Klercker, *Hormidium subtile* (Kützing) Heering, *Chlorhormidium subtile* (Kützing) Starmach, *Ulothrix subtilissima* Rabenhorst, *Ulothrix subtilis* var. *subtilissima* (Rabenhorst) Rabenhorst, *Ulothrix subtilis* spp. *subtilissima* (Rabenhorst) Hansgirg, *Hormiscia subtilis* (Kützing) De Toni, *Hormiscia subtilis* var. *subtilissima* (Rabenhorst) Hansgirg, *Hormidium subtilissimum* (Rabenhorst) Mattox & Bold, *Chlorhormidium subtilissimum* (Rabenhorst) Fott, *Klebsormidium subtilissimum* (Rabenhorst) P.C.Silva, Mattox & Blackwell, *Klebsormidium subtile* (Kützing) Tracanna ex Tell nom. inval.

*Type locality*: In a mill‐course.

*Emended description*: Filaments long, with some tendency to fragmentation, sometimes curved, thin or medium in width, in young culture cells are long and cylindrical, in mature and old cultures sometimes isodiametric, (5.1)6.0–6.6(7.0) × (4.7)5.8–10.3(11.5) μm (length/width—1.0–1.8), filaments are slightly bead‐like, constricted near cross walls, H‐like pieces of cell walls sometimes present; chloroplast covering 2/3 of the cell inner surface, with smooth or undulating margins; the pyrenoid is small, round, compact, surrounded by a layer of starch grains. It forms submerged tufts and a superficial layer in liquid culture, and smooth or slightly undulating colonies on an agar plate.

*Epitype (designated here)*: Strain SAG 384‐1 (proposed here as the authentic strain of *K. subtile*) is permanently preserved in a metabolically inactive state (cryopreserved in liquid nitrogen) in the SAG.

*Comments*:* K. subtile* is morphologically and ecologically close to *K. subtilissimum* (Rabenhorst) P.C. Silva, Mattox et Blackwell. Both these taxa may represent a single species inhabiting water bodies or humid terrestrial habitats. They were initially described as representatives of *Ulothrix* Kützing (Kützing 1845, Rabenhorst 1857). As *K. subtile* was described earlier than *K. subtilissimum* (as *Ulothrix subtilis* Kützing 1845), the former name has priority. Strain SAG 384‐1, initially identified as *K. subtilissimum*, is proposed as the epitype for *K. subtile*. Strain SAG 384‐1 was isolated by R.A. Lewin from snow (United States). It does not conflict with the original description (Kützing 1845), the description in Tell (1976) and thus generally corresponds to the emended description. The combination *Klebsormidium subtile* (Kützing) Tracanna ex Tell (Tell 1976, p. 535) is invalid because although the basionym is listed in this study, a complete and direct reference to its author and place of valid publication including page reference and date is missing (see Article 41.5 of the ICN (McNeill et al. 2012)).

*Klebsormidium nitens* (Kützing) Lokhorst (1996). *Cryptogamic Studies* 5:13–17, figs. 1–30.

*Basionym*:* Ulothrix nitens* Kützing (1849: 349). Species Algarum.

*Synonyms*: see Lokhorst (1996:13–14).

*Type locality*: In plate with another terrestrial alga *Palmella cruenta* (J.W. Smith) C. Agardh collected in Italy.

*Lectotype*: L 939.67‐828, annotated as “Cum Palmella cruenta in cubicula culta,” leg. G. Meneghini, designated by Lokhorst (1996).

*Epitype*: Strain SAG 13.91, designated here to support the lectotype specified above. This authentic strain of *K. nitens* is permanently preserved in a metabolically inactive state (cryopreserved in liquid nitrogen) in the SAG.

*Comments*: The *K. nitens* morphotype is present in several lineages of clade E (Fig. 1), but most of the investigated strains with this morphotype can be grouped in E2 (Rindi et al. 2011, Škaloud and Rindi 2013). Strains of clade E2 were proposed as good candidates for designation as *K. nitens* (Škaloud et al. 2014). We propose as the epitype strain SAG 13.91 that belongs to E2 (Rindi et al. 2011), and its morphological and ecological features correspond with the diagnosis of *K. nitens* as emended by Lokhorst (1996). SAG 13.91 was isolated by E.A. Flint from Tekoa soil (New Zealand), original number No 60/74. It does not conflict with the original description (Kützing 1849) and generally corresponds to the emended description. SAG 13.91 is also genetically close to Lokhorst's strain of *K. nitens* (KL 37), which was lost (Rindi et al. 2011).

*Klebsormidium dissectum* (F. Gay) H. Ettl et G. Gärtner (1995). Syllabus der Boden‐, Luft‐ und Flechtenalgen:601.

*Basionym*:* Stichococcus dissectus* Gay (1891). Recherches dévelop. classific. algues vertes: 78, pl. X: figs. 96–97, pl. XI: 98–100.

*Synonyms*:* Klebsormidium dissectum* (F. Gay) Lokhorst nom. inval., *K. dissectum* (F. Gay) T. Mrozinska nom. inval., see also Lokhorst (1996: 24).

*Emended description*: Lokhorst (1996) Cryptogamic Studies 5: 24–25, figs. 110–129.

*Neotype*: prepared from cultures initiated from a soil sample collected from a forest track near Col du Bussang (France). 12.09.1992, leg. Lokhorst (L), designated by Lokhorst (1996).

*Epitype*: Strain SAG 2417 is designated here to support the neotype specified above and proposed as the authentic strain of *K. dissectum*. It is permanently preserved in a metabolically inactive state (cryopreserved in liquid nitrogen) in the SAG.

*Comments*: Strain SAG 2155 isolated by Lokhorst from the neotype locality (original number KL 2) cannot be used as the epitype, because it does not correspond to emended description of *K. dissectum*. SAG 2155 morphologically and genetically corresponds to *K. nitens* (Rindi et al. 2011). Perhaps, this strain was mislabeled. We propose a strain SAG 2417 that was isolated by U. Karsten (originally labeled BOT2) from the concrete basement of a greenhouse in the Botanical Garden of Innsbruck (Austria) as the epitype of *K. dissectum*. It does not conflict with original description (Gay 1891) and generally corresponds to the emended description (Lokhorst 1996). It differs in having slightly thinner filaments, reaching 8.6(9.3) μm (in emended description—to 9.3(10.2) μm). The taxonomic combination *Klebsormidium dissectum* (Gay) Lokhorst (1996) mentioned by Lokhorst (1996, p. 24) is invalid because of priority rules.

*Klebsormidium fluitans* (F. Gay) Lokhorst (1996). *Cryptogamic Studies* 5:20–23, figs. 69–109.

*Basionym*:* Stichococcus fluitans* Gay (1894) Bull. Soc. Bot. France 40: CLXXIV, fig. 1.

*Synonyms*: see Lokhorst (1996: 20).

*Emended description*: Lokhorst (1996) Cryptogamic Studies 5: 20–23, figs 69–109.

*Neotype*: prepared as a herbarium sheet from a former culture derived from material collected from sheet‐piling of Westeinderplassen Lakenear Rijsenhout (the Netherlands), 17.11.1992, leg. Lokhorst (L), designated by Lokhorst (1996).

*Epitype*: Strain SAG 9.96, designated here to support the neotype specified above and proposed here as the authentic strain of *K. fluitans* that is permanently preserved in a metabolically inactive state (cryopreserved in liquid nitrogen) in the SAG.

*Comments*: Strain SAG 9.96 was isolated by Lokhorst from the neotype locality (original number KL 22). It does not conflict with the original description (Gay 1894) and generally corresponds to the emended description. It differs in the absence of pseudobranching in the current state of culture, and in having a prominent starch envelope composed of several layers of small starch grains surrounding the pyrenoid (the starch envelope of the pyrenoid is visible in Lokhorst's micrographs 102–104, although the description states: “without starch envelope”).

*Klebsormidium mucosum* (J.B. Petersen) Lokhorst in Lokhorst and Star (1985). *J. Phycol*. 21:474.

*Basionym*:* Hormidium mucosum* Petersen (1915). K. Dan. Vidensk. Selsk. Biol. Skr. 7. Raekke, Naturv. Math. 12: 340, 376, text‐figs. 21, 22, pl. III: figs. 38–40.

*Synonyms*: see Lokhorst (1996:34).

*Emended description*: Lokhorst (1996) Cryptogamic Studies 5: 34–37, figs. 220–254.

*Type locality*: On naked clay soil of road in plantation at Rø, Bornholm (Denmark).

*Lectotype*: C, annotated as “*Hormidium mucosum* n. sp. Road in plantation at Rø, Bornholm, on naked clay soil, 12.10.1912”; designated by Lokhorst and Star (1985).

*Epitype*: Strain SAG 8.96 designated here to support the lectotype specified above and is the authentic strain of *K. mucosum*, that is, permanently preserved in a metabolically inactive state (cryopreserved in liquid nitrogen) in the SAG.

*Comments*: Strain SAG 8.96 was isolated by Lokhorst (original number KL 63) from soil near the water level of the river Dommel near Valkenswaard (the Netherlands). It does not conflict with the original description (Petersen 1915) and generally corresponds to the emended description. The only difference is the slightly thinner filaments, not exceeding 20.0 μm (in original description—up to 23.3 μm).

*Klebsormidium elegans* Lokhorst (1996). *Cryptogamic Studies* 5:28–30, figs. 149–180.

*Heterotypic synonym*:* K. bilatum* Lokhorst

*Original description*: Lokhorst (1996) Cryptogamic Studies 5: 28–30, figs. 149–180.

*Holotype*: prepared from a herbarium sheet of a former culture derived from an algal coat growing on bark on the foot of an oak tree, near Staverden (the Netherlands). 24.11.1992, leg. Lokhorst. (L), designated by Lokhorst (1996).

*Epitype*: Strain SAG 7.96 designated here to support the type specified above and proposed as the authentic strain of *K. elegans* is permanently preserved in a metabolically inactive state (cryopreserved in liquid nitrogen) in the SAG.

*Comments*: Strain SAG 7.96 was isolated by Lokhorst from the type locality (original number KL 24) and generally corresponds to the original description. The only difference consists of the slightly thinner filaments—(7.8)8.1–9.3(10.0) μm (in original description—(8.4)9.3–10.2(13.0) μm). *Klebsormidium bilatum* is proposed as a synonym of *K. elegans* because the ITS and *rbc*L sequences as well as the morphological characters of the strain isolated by Lokhorst (SAG 5.96) are close to the proposed epitype strain (see Rindi et al. 2011 and present paper). The *rbc*L sequences of both strains (SAG 7.96 and SAG 5.96) are identical, but ITS 2 sequences have differences in one nucleotide.

### Other species of *Klebsormidium*, poorly described or doubtful taxa

The remaining species of *Klebsormidium* are morphologically similar to the above‐mentioned taxa, and hence may be synonyms of these, but need additional research. *Klebsormidium klebsii* (G.M. Smith) P.C. Silva, Mattox & Blackwell is probably a synonym of *К. flaccidum*;* K. lamellosum* Y.X.Wei & H.Hu and *K. montanum* (Hansgirg) S. Watanabe are morphologically similar to *К. crenulatum*;* K. sterile* (Deason & Bold) P.C. Silva, Mattox & Blackwell is close to *K. nitens*. Species of *Klebsormidium* with extremely thin filaments (*K. pseudostichococcus* (Heering) H. Ettl & G. Gärtner, *K. tribonematoideum* (Skuja) Hindák and *K. scopulinum* (Hazen) H. Ettl & G. Gärtner) probably belong to the genera *Stichococcus* Nägeli or *Gloeotila* Kützing (Lokhorst 1996). Another species with thin filaments (*K. marinum* (Deason) P.C. Silva, Mattox & Blackwell) was transferred to *Stichococcus* as *S. deasonii* Neustupa et al. (2007). *Klebsormidium drouetii* H.P. Wagner & J.S. Zaneveld and *K. rivulare* (Kützing) M.O. Morison & Sheath are insufficiently described and doubtful taxa, and the latter may be a species of *Ulothrix* (Lokhorst 1996). *K. fragile* (Kützing) H.P. Wagner & J.S. Zaneveld and *K. catenatum* (Dangeard) Guiry are invalidly described taxa (http://ucjeps.berkeley.edu/CPD; Guiry and Guiry 2015). Different intraspecific taxa of *K. flaccidum* are now restricted to synonyms of morphologically related species: f. *aquatica* (Heering) Nizamuddin & Gerloff to *K. mucosum*, f. *tumidum* (Heering) H. Ettl & G. Gärtner to *K. dissectum*, var. *crassum* (Chodat) H. Ettl & Gärtner to *K. flaccidum*, and var. *lubricum* (Chodat) H. Ettl & G. Gärtner to *K. nitens* (Lokhorst 1996).

Our sincere thanks to Dr. Thomas Pröschold for providing sequences of the alpine *Klebsormidium* strains and fruitful discussion on taxonomic aspects, to Prof. Thomas Friedl for making it possible to compare our material with strains in the SAG collection, and to Dr. Maike Lorenz and the SAG staff for their help with cryopreservation of epitype strains. T.M. thanks the DAAD for a short‐term research fellowship and the Alexander von Humboldt Foundation for a Georg‐Forster research fellowship at the University of Rostock. The support of this study by Austrian Science Fund (FWF) grant P 24242‐B16 is acknowledged by A.H. Finally, U.K. thanks the DFG for financial support (KA899/16‐1/4) and the University of Innsbruck as host during his sabbatical.

## Supporting information


**Table S1.** Information for *Klebsormidium* strains isolated from alpine soil crusts in the Tyrolean Alps, Austria, and Italy.
