# Supplementary material for: Biodiversity of Klebsormidium (Streptophyta) from alpine biological soil crusts (Alps, Tyrol, Austria, and Italy)
Source: J Phycol. 2015 Jul 14;51(4):750–67. doi: 10.1111/jpy.12316 (PMC4618304; doi:10.1111/jpy.12316)
Supplement: Supplementary file 1 — Table S1. Information for Klebsormidium strains isolated from alpine soil crusts in the Tyrolean Alps, Austria, and Italy. [file JPY-51-750-s001.doc]

**Supplementary Table S1**

Information for *Klebsormidium* strains isolated from alpine soil crusts in the Tyrolean Alps, Austria and Italy

| Original number of strain (number in culture collection) | Identification | Origin | Genbank accession number (ITS rDNA) |
| --- | --- | --- | --- |
| AH1 (SAG 2415) | *K. crenulatum* | 46° 50.998’ N, 11° 00.903’ E; 2350 m a.s.l., soil crust at Schönwieskopf, Obergurgl, Austria, isolated July 2007 by Angelika Tschaikner & Andreas Holzinger | HG973040 |
| AH2 (SAG 2416) | *K.* cf. *nitens* | The same information | HG973020 |
| OBG | *K.* cf. *nitens* | The same locality, isolated 6^th^ Apr. 2009 by Ulf Karsten | HG973024 |
| ASIB V100 | *K. flaccidum* | 46° 34.510’ N, 11° 43.280’ E; 2363 m a.s.l., soil crust under *Festuca rubra*, Pitschberg, St. Ulrich in Gröden, South Tyrol, Italy, isolated Oct. 1969 by Georg Vinatzer | HG973008 |
| ASIB V103 | *K. fluitans* | The same information | HF955434 |
| ASIB V108 | *K. fluitans* | The same information | HG973019 |
| VOE2 | *K. fluitans* | 47° 14.839’ N, 11° 19.804’ E; 649 m a.s.l., soil crust in pine forest gully, Völs, Innsbruck, Austria, isolated 6^th^ Apr. 2009 by Ulf Karsten | HG973026 |
| VOE3 | *K. fluitans* | The same information | HG422825 |
| VOE4 | *K. fluitans* | The same information | HG973028 |
| VOE5 | *K. subtile* | The same information | HF955435 |
| VOE7 | *K. fluitans* | The same information | HG973029 |
| VOE8 | *K. fluitans* | The same information | HG973027 |
| BOT1 | *K.* cf. *fluitans* | 47° 16.075’ N, 11° 22.718’ E; 609 m a.s.l., crust on concrete basement of old greenhouse in the Botanical Garden, University of Innsbruck, Austria, isolated 21^th^ Apr. 2009 by Ulf Karsten | HG973030 |
| BOT2 (SAG 2417) | *K. dissectum* | The same information | HG973017 |
| BOT3 | *K.* cf. *fluitans* | The same information | HG422826 |
| BOT4 | *K. dissectum* | The same information | HG973018 |
| RAU1 | *K. nitens* | 47° 12.242’ N, 11° 11.581’ E; 1074 m a.s.l., soil crust, pine forest, Rauth, Sellrain Valley, Austria, isolated 27^th^ Apr. 2009 by Ulf Karsten | HG973038 |
| RAU2 | *K. subtile* | The same information | HG973031 |
| RAU3 | *K.* cf. *fluitans* | The same information | HG422828 |
| SIE1 | *K.* cf. *flaccidum* | 47° 11.908’ N, 11° 5.901’ E; 1548 m a.s.l., soil crust, pine forest, St. Siegmund, Sellrain Valley, Austria, isolated 27^th^ Apr. 2009 by Ulf Karsten | HG973013 |
| SIE2 | *K.* cf. *subtile* | The same information | HF955436 |
| SIE3 | *K.* cf. *flaccidum* | The same information | HG973014 |
| STR1 | *K. nitens* | 47° 20.189’ N, 11° 1.738’ E; 1280 m a.s.l., soil crust, pine forest, Strassberg Hut, Austria, isolated 3^rd^ May 2009 by Ulf Karsten | HG973010 |
| STR2 | *K.* cf. *subtile* | The same information | HG973021 |
| STR3 | *K.* cf. *subtile* | The same information | HG973022 |
| OBE1 | *K. nitens* | 47° 26.459’ N, 12° 2.102’ E; 1046 m a.s.l., soil crust, pine forest, Oberau, Wildschönau, Austria, isolated 21^th^ May 2009 by Ulf Karsten | HG422827 |
| OBE2 | *K. nitens* | The same information | HG973032 |
| KUE1 | *K.* cf. *flaccidum* | 47° 13.843’ N, 11° 0.840’ E; 2435 m a.s.l., soil crust, Limnological Station Gossenköllsee, University Innsbruck, Austria, isolated 3^rd^ Jun. 2009 by Ulf Karsten | HF955437 |
| KUE2 | *K.* cf. *flaccidum* | The same information | HG973016 |
| PAT | *K.* cf. *nitens* | 47° 12.582’ N, 11° 28.006’ E; 2145 m a.s.l., soil crust, Patscherkofel, Innsbruck, Austria, isolated 1^st^ Jun. 2009 by Ulf Karsten | HG973033 |
| BIR | *K. subtile* | 47° 12.011’ N, 11° 18.848’ E; 1953 m a.s.l., soil crust, Birgitzer Alm, Innsbruck, Austria, isolated 13^th^ Jun. 2009 by Ulf Karsten | HG973036 |
| STU1 | *K.* cf. *flaccidum* | 46° 59.205’ N, 11° 6.953’ E; 2866 m a.s.l., soil crust on rocks, Schaufelferner, Stubai Valley, Austria, isolated 3^rd^Jul. 2009 by Ulf Karsten | HG973015 |
| STU3 | *K. subtile* | The same information | HG973023 |
| MUT | *K.* cf. *subtile* | 46° 50.903’ N, 11° 1.844’ E; 2650 m a.s.l., soil crust at Hohe Mut, Obergurgl, Austria, isolated 9^th^ Jul. 2009 by Ulf Karsten | HG973034 |
| HOH1 | *K. subtile* | 46° 53.861’ N, 10° 38.364’ E; 2207 m a.s.l., soil crust, Hohenzollernhaus, Radurschl Valley, Austria, isolated 13^th^ Jul. 2009 by Ulf Karsten | HG973035 |
| HOH2 | *K. subtile* | The same information | HG973011 |
| PIT1 | *K. bilatum* | 46° 55.609’ N, 10° 52.745’ E; 2843 m a.s.l., soil crust on rocks, Pitz Valley Glacier, Austria, isolated 22^th^ Jul. 2009 by Ulf Karsten | HG973012 |
| PIT2 | *K. subtile* | The same information | HG973037 |
| PIT3 | *K. bilatum* | The same information | HG973039 |
| UEB | *K. nitens* | 47° 9.032’ N, 13° 52.132’ E; 1680 m a.s.l., soil crust, pine forest, Tamsweg, Lungau, isolated 26^th^ Jun. 2009 by Ulf Karsten | HG973025 |
